# President’s Address

**Published:** 1897-09

**Authors:** A. W. Diack


### THE DENTAL REGISTER.

VOL. LI.] SEPTEMBER, 1897. [No. 9.

### COMMUNICATIONS.

### President's Address.

BY DR. A. W. DIACK, D.D.S.

Read before the Michigan State Dental Society, June 14th, 1897.

Although in previous years, I believe, the annual address of the President was dispensed with—without any great loss to the members of the society, it was thought by the Board of Directors that the President should this year be placed upon the program for an annual address. It is in pursuance of this idea of the board that I am now before you to offer what to my mind ought to be brought before you for consideration, in fact, I have so far framed what I am going to say, so that it will embody the results accomplished by this society during the past year, and also the work which it should accomplish during the next year, and to make some suggestions for committee action so that this work can be facilitated.

To begin, it will be well to glance at the status of the dental profession throughout the State so that we may be better able to grasp the situation. We have, according to the records of the State Board of Examiners, some 700 practitioners in the State—of these only about one-fourth are members of the society. The profession of the State as represented by this society—and it is no fault of the society that it is not better represented, has succeeded in maintaining a high standard of education in the State by means of legislative acts. This has without doubt had great weight in the opinion of the public concerning the high standing to which the dental profession has attained—so that to-day we find that while the medical profession is suffering sadly from the lack of sufficiently high standards, the dental profession is firmly fixed so that it may reap the benefits of its position by having its members assured of a thorough education in its special branches.

But when we consider the relative proportion which the members of the society bear to the total number of practitioners, it is very soon made apparent that all the work necessary to the accomplishment of the profession has been done by a very small number, while the majority have shared the benefit and aided not at all. This is not as it should be and some means should be taken to show the non-members their indebtedness to the society, how vital to their personal interests its existence is, and how necessary it is that all honest men should co-operate for the enlargement and strengthening of the society.

As to how this should best be accomplished, it is hard to say, but I would suggest as a first step to a more efficient organization the following scheme:

Have the officers of the State society encourage by such means as it has at hand the formation of local societies in all parts of the State. I would suggest to the society the plan of dividing these parts of the State which have at present no local societies, into districts of such size as is thought best, keeping in mind the facilities for meeting, and notify all dentists within the district of the plan. It could then be left entirely in the hands of the local men who could carry on the work in their own way. Then when new organizations were formed, it would be well to institute a method of communication from the local societies with the State, so that there would be some tangible connection between the two, and thus the whole would be organically inter-dependent. It would only consume time were I to enter into a discussion of the present relation of the local and State societies—suffice it to say that there is a total separation of the two.

That some such organization is necessary is seen whenever there is to be any action taken outside of a scientific sphere. Especially is it so when we wish to accomplish anything with the legislature, where a full representation is of the utmost importance. I sincerely hope that some such plan of organization can be perfected as it will eventually result in the general advancement of the profession.

Before proceeding further it would be well to call attention of the society to the preventive measures which were taken in the matter of adverse dental legislation in the last session. By taking the matter in time and notifying all members of the House and Senate, by means of letters setting forth the facts of the case, and also letters to all members of the society, urging personal effort with their representatives, the subject of dental legislation was wholly discouraged and we have again reason for self-congratulation in that our dental law is still in force. In a paper in the program the subject of the enforcement of the law will be thoroughly discussed and it is to be hoped that all efforts at such enforcement will meet with support from the society.

Another thing which ought to be brought to the attention of the society at the present time is the tendency, which has been noticed during the last two or three years, to resort to unethical practices in the offices of many of the recent graduates and the general lack of tone in the morality of practice in the profession at large. It is needless to point out the futility of resorting to advertising as a means of establishing a practice as even a most obtuse observer can see that the "day of the advertiser is short in the land." But needless as it may seem to mention it before the society, it should be expressed as the opinion of the society, and preached as such, that such practice is not only detrimental to all true and honest advancement in science, but is indeed a most effective obstacle to the establishment of a practice which can be depended upon. This idea should be brought more forcibly to the minds of all entering the practice, and the State society should stand pre-eminently as the disciple of honest and ethical practice.

In these days of competition, not only between the members of the profession but even of the colleges which teach dentistry, it is no wonder that many of the new graduates rush at once into advertising and justify themselves by the thought that older practitioners do it, or that their college, their alma mater, was in the habit of advertising in the public prints for patients for its infirmary, and then as the graduate well knew, charging almost the usual office price for the work which was done. When such practices are indulged by the colleges of the present day is it any wonder I ask that the men who graduate from these colleges do likewise? It will indeed be a symptom of health in a diseased body when the National Association of Dental Faculties sees fit to apply the code of dental ethics to its colleges with the same spirit in which the societies apply it, and I recommend that resolutions be passed to this end.

Another unhealthful sign is the appearance in the advertising columns of several dental journals of advertisements of the professional advertisement writer. These "ads," appearing as they have done, in the columns of journals which have heretofore stood high in the literature and which depend almost entirely for their reading matter upon the societies, should be discouraged as much as possible, and if it is possible it would perhaps be a wholesome rebuke to have this society pass a resolution condemning such practice. If the honor and advancement of dentistry is to be preserved the dental society must be preserved, and no society dependent upon voluntary mutual service of its members is going to succeed upon other lines than ethical ones, and it behooves all honest men in the profession to rise against the insidious practices which are gradually undermining the manliness of our profession. I sincerely hope that some action will be taken on these lines.

During the year past, the dental society had occasion to mourn the death of two of its members—Dr. Jere Robinson and Dr. W. H. Ray. It is no exaggeration to say that the present status of our profession is due to the honest manhood, the noble aspirations and the self-sacrificing brotherhood of these two men, whose absence from our midst to-day, will be marked in the heart of many with a feeling of sadness.

---

EQUAL parts of glycerine and tincture of iodine, applied every day or two to the diseased mucous membrane in cases of granular conjunctivitis will cause the membrane to present a smooth and healthy appearance.

